# Addressing Linkage Errors in the New Zealand Integrated Data Infrastructure: Characterization, Mitigation, and Implications.

**DOI:** 10.23889/ijpds.v9i5.2720

**Published:** 2024-09-18

**Authors:** Eileen Li, Barry Milne

**Affiliations:** 1 University of Auckland

The New Zealand Integrated Data Infrastructure (IDI) is a database of de-identified linked whole population administrative records, which is available for research under strict access conditions. Within the IDI, individual records undergo probabilistic linkage and are subject to linkage errors. Despite this, most research conducted using IDI data operates under the assumption of perfect linkage, neglecting the potential impact of these errors on analytical outcomes. Our study aims to address this gap by identifying and characterizing the two types of linkage errors – false links and missed links – utilizing data from two IDI datasets: New Zealand Census 2013 and Education. We implement approaches to demonstrate the effect of linkage errors and adjust for them to mitigate the influence of linkage errors on modelling results. Through a practical demonstration, we illustrate how false links and missed links can affect a simple regression model. Our findings reveal that while false links and missed links do introduce biases into modelling results, the magnitude of these biases is often within acceptable limits. However, as the cumulative effect of errors increases with the addition of more data sources, there is a potential for practically significant changes in modelling outcomes. This underscores the importance of acknowledging and addressing linkage errors in linked data analysis particularly for those utilizing IDI data, as failure to do so can lead to misleading conclusions. Our study highlights the necessity of accounting for linkage errors in research and policy-making processes to ensure robust and reliable findings.

